# Introduction of the special issue on COVID-19 and the financial and economic systems

**DOI:** 10.1186/s40854-022-00363-4

**Published:** 2022-05-12

**Authors:** Paresh Kumar Narayan

**Affiliations:** Monash Business School, Monash University, Melbourne, Australia

A large number of submissions were received in response to this call for paper on the theme of COVID-19 and the financial and economic systems. Naturally, the COVID-19 pandemic has dominated much of the recent finance and economics literature. This call for paper was motivated by early special issues on the COVID-19 business and economics themes such as those published by Sha and Sharma (2020) and Sharma and Sha (2020, 2021). This editorial has two parts. In the first part I summarize the key outcomes from each of the papers. In the second part, I identify an agenda for future research that has emanated from this special issue papers.

## I. A summary of the special issue papers

There are a total of 13 papers in this 32nd volume of Financial Innovation (FIN), Volume 8, No.2 (2022). Papers in this special issue can be grouped into three sub-themes. The first sub-theme is the stock market reaction to the COVID-19 pandemic. This is a popular subject and therefore having the bulk of papers on this sub-theme is unsurprising. Hong, Bian and Lee test the effect of structural breaks in the relation between COVID-19 and the US stock market. They discover a break that corresponds to the onset of the pandemic. The authors draw implications for traders. COVID-19 and sectoral returns were studied by Shahzad, Bouri, Kristoufek and Saeed who show evidence of intensified connectedness in sectoral returns around the pandemic. Hui and Chan add to these studies by evaluating the effects of the pandemic on global equity markets and show a negative effect. Wang and Liu show that the effect of the pandemic on the stock market is heterogenous—that is, it is industry-specific—a finding consistent with He et al. (2020). The financial market effects of volatility spillovers for cryptocurrency returns were studied by Özdemir and were shown to be more pronounced during the second COVID-19 lockdown starting at the beginning of November 2020. The stock market—economic policy uncertainty effect due to the pandemic was studied by Dai, Xiong, Liu, Huynh and Sun and is consistent with the evidence in Iyke (2020). The authors show the manner in which EPU impacts the stock market was heightened due to the pandemic.

The second sub-theme is the effect of the pandemic on commodities: Zhang, Narayan and Devpura examine how COVID-19 changed the stock returns-oil price predictability relationship. More specifically, their empirical analysis shows that the effect of oil price on stock returns declined by around 89.5% due to the COVID-19 pandemic. Salisu and Obiora studied the hedging effectiveness of financial innovations against crude oil investment risks. They find higher hedging performance during the pandemic and the authors draw implications for investors from these results. Maghyereh and Abdoh examine whether news-based economic sentiment predicts bubbles in the precious metals market. They find they do.

The third group of studies is relatively more innovative and newer by way of research hypotheses. For instance, Narayan, Rizvi and Sakti propose and evaluate the role of green investments on portfolio diversification and risks. They show that Sukuk and green Sukuk offer strong potential for diversification during the pandemic. Liébana‐Cabanillas, Muñoz‐Leiva, Molinillo and Higueras-Castillo evaluate whether biometric payment systems work during the pandemic. Nigmonov and Shams show that the probability of default increased due to the pandemic. Borrowers with lower credit ratings and those from countries with weal FinTech innovations were more severely impacted. Specific events resulting directly out of the pandemic, such as the light a lamp event in India were linked to the stock market behavior by Chundakkadan. He shows that the Indian stock market rose by 9% post this event.

## II. Implications for future research

The first subset of papers in this special issue on the effects of the pandemic on the stock market, because they use more recent data than previous studies (see Phan and Narayan 2020; Sharma and Sha 2020, 2021; Sha and Sharma 2020 and Yang and Deng 2021) merely provide a robustness test to earlier hypotheses tests. This is welcomed because one limitation of the earlier studies on COVID-19 has been the lack of data (see Narayan 2021). One direction for future studies that remains is to evaluate more the industry or sectoral links to the pandemic and understand the dynamic effects, effects on value chains, understanding supply chain bottlenecks and constraints in terms of human resources and digitalization aspects of industrial growth during the pandemic. None of these issues have been explored in-depth and there is scope for new research on these business and economic aspects.

The second subset of studies published in this issue relating to the commodities market deserves more works along the following lines. First, the co-existence of the pandemic with falling oil prices initially and then a significant growth in oil prices with the on-set of geopolitical tensions marks an interesting platform on which to study the three-way relationship between the financial system, and geopolitical tensions (trade wars included) and oil prices. Theoretical models that identify the role of trade wars in economic growth and corporate growth/performance will be needed to enhance research in this area. In this regard, the story of oil price and the pandemic has already been discussed in Devpura (2020), Devpura and Narayan (2020), Prabheesh et al. (2020), and Wang and Su (2021).

The third group of studies occupying this special issue motivates several lines of additional research. First, the issue of news sentiment is important from behavioral finance and economic points of view in understanding the effects of the pandemic on the financial and economic system. This stands out to be an area that deserves greater research attention. Fittingly, new news-based datasets have been developed, such as the one by Narayan et al. (2021). This dataset has news on several variants of the pandemic and would therefore be useful in studying the effects of the pandemic (from a heterogeneous perspective) on the financial and economic systems. Some studies have already analyzed the role of news during the pandemic; see Hoang and Syed (2021) and Salisu and Akanni (2020).

Second, more studies are needed to evaluate the effects of specific events resulting from the pandemic on the financial economic systems. This will allow us to understand the relevance of policies in mitigating the effects of the pandemic. Some of the events about whose effectiveness we still do not fully understand are the (a) roles of specific fiscal and monetary policies; (b) geographic lockdown effects; and (c) digital trade. For some preliminary studies, see Phan and Narayan (2020), and Yang and Deng (2021).

Third, there is limited work on COVID-19 related portfolio diversification as conducted in this special issue by Narayan et al. and portfolio-based investments such as Prabheesh (2020). This deserves more research to understand the relevance and effectiveness of different trading strategies during the pandemic across a wide range of assets.
